# *Tauritermes bandeirai*: A new drywood termite (Isoptera, Kalotermitidae) from the Caatinga and Atlantic Forest of Brazil

**DOI:** 10.3897/zookeys.954.52335

**Published:** 2020-07-29

**Authors:** Rudolf H. Scheffrahn, Alexandre Vasconcellos

**Affiliations:** 1 Fort Lauderdale Research and Education Center, University of Florida, 3205 College Avenue Davie, Florida 33314, USA University of Florida Florida United States of America; 2 Universidade Federal da Paraíba, Departamento de Sistemática e Ecologia, Laboratório de Termitologia, CEP: 58051-900, João Pessoa, Paraíba, Brazil Universidade Federal da Paraíba Paraíba Brazil

**Keywords:** Imago, new species, soldier, South America

## Abstract

The imago and soldier castes of a new *Tauritermes* Krishna, 1961 species, *Tauritermes bandeirai***sp. nov.** are described. It is the fourth species of *Tauritermes* and occurs from the Caatinga and Atlantic Forest of Brazil. Unlike its congeners, the soldier of *T. bandeirai* has prominent frontal horns.

## Introduction

In South America, six kalotermitid genera have soldiers with partial to robust head capsule phragmosis: *Calcaritermes*, *Cryptotermes*, *Eucryptotermes*, *Glyptotermes*, *Proneotermes*, and *Tauritermes* (Scheffrahn 2019a). According to Krishna et al. (2013), *Tauritermes* Krishna, 1961 was known only from southern Brazil and northern Argentina. Actually, Mélo and Bandeira (2004) and Vasconcellos et al. (2005) reported an unidentified species from the semiarid Caatinga and Atlantic Forest of northeastern Brazil, respectively. This was followed by additional *Tauritermes* records from the same region (Vasconcellos et al. 2010; Souza et al. 2012; Cancello et al. 2014). Dambros et al. (2013) expanded the range of *Tauritermes* to include Amazonas (Manaus). Most recently, Scheffrahn (2019a) reported *Tauritermes* from Bolivia and Paraguay.

*Tauritermes*, as other kalotermitid genera, is best characterized by venation of the winged imago and soldier head capsule morphology. For Kalotermitidae, most diagnostic characters at the intrageneric level are attributed to head capsule characters. Noirot (1995) reported some consistent differences in the gut anatomy of kalotermitids compared to other lower termite families. Furthermore, Gonçalvez (1979), Myles (1997), and Godoy (2004) described parts of the gut of *Rugitermes*, *Marginitermes*, and *Tauritermes*, respectively. They found that differentiation of the gut morphology of kalotermitids remains elusive at both the generic and specific levels.

Herein, we report on a new *Tauritermes* species, *T. bandeirai* sp. nov. from samples collected by Vasconcellos et al. (2005) and additional specimens from the Brazilian Caatinga and Atlantic Forest. This is the fourth species of *Tauritermes* to be described based on external imago and soldier characters.

## Material and methods

Photomicrographs were taken as multi-layer montages using a Leica M205C stereomicroscope controlled by the Leica Application Suite version 3 software. Preserved specimens were taken from 85% ethanol and suspended in a pool of Purell Hand Sanitizer to position the specimens on a transparent Petri dish background. All University of Florida Termite Collectio (UFTC) records are available online (Scheffrahn 2019b).

## Taxonomy

### Tauritermes bandeirai sp. nov.

Taxon classificationAnimaliaBlattodeaKalotermitidae

B7F2B6C0-C7BA-5756-8D6F-D5F0E629B0C6

http://zoobank.org/3C739327-4FB8-4B77-8AD0-862A82237292

Figures 1 , 2

#### Diagnosis.

The soldier of *T. bandeirai* differs from soldiers of the other three *Tauritermes* species by having a distinct and robust frontal horn and a roundly protruding dorsal horn (Fig. 2C). The dorsal and frontal horns of *T. triceromegas* (Silvestri, 1901) are more angular but much smaller, barely elevated above the frons in oblique view (Fig. 3C). In *T. vitulus* Araujo & Fontes, 1979, the dorsal horn is similar to that of *T. bandeirai* sp. nov., while the fronal horn is absent (Araujo and Fontes 1979, fig. 10). In *T. taurocephalus* (Silvestri, 1901), the dorsal horn is more elevated and angular, while the frontal horn is also absent (Fig. 4C).

**Figure 1.**
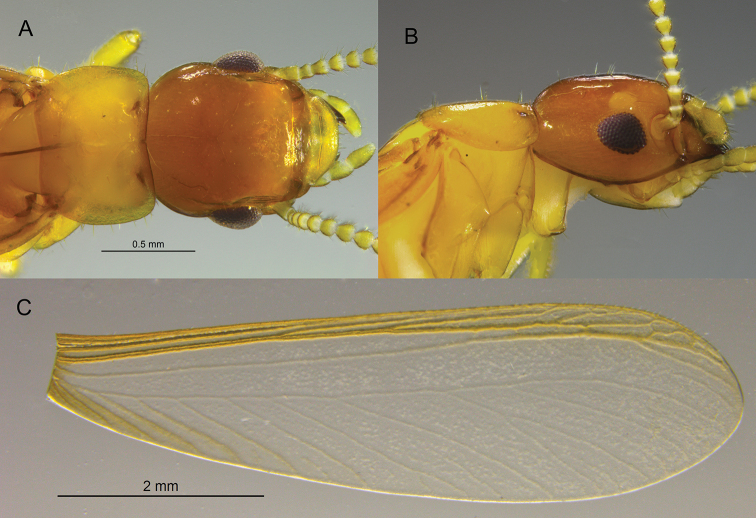
Imago of *Tauritermes bandeirai* sp. nov. (SA502) **A** dorsal and **B** lateral views of head and pronotum **C** right forewing.

**Figure 2.**
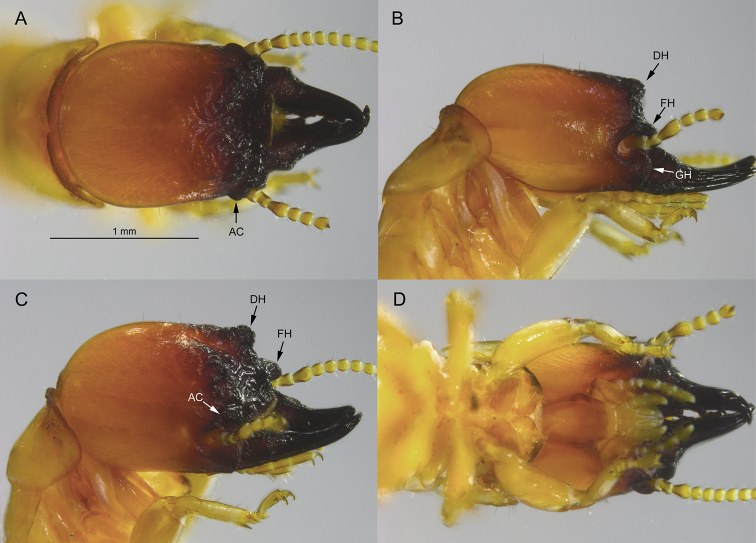
Soldier of *Tauritermes bandeirai* sp. nov. (SA499) **A** dorsal **B** lateral **C** oblique, and **D** ventral views of head and pronotum. AC = antennal carina, DH = dorsal horn, FH = frontal horn, and GH = genal horn.

**Figure 3.**
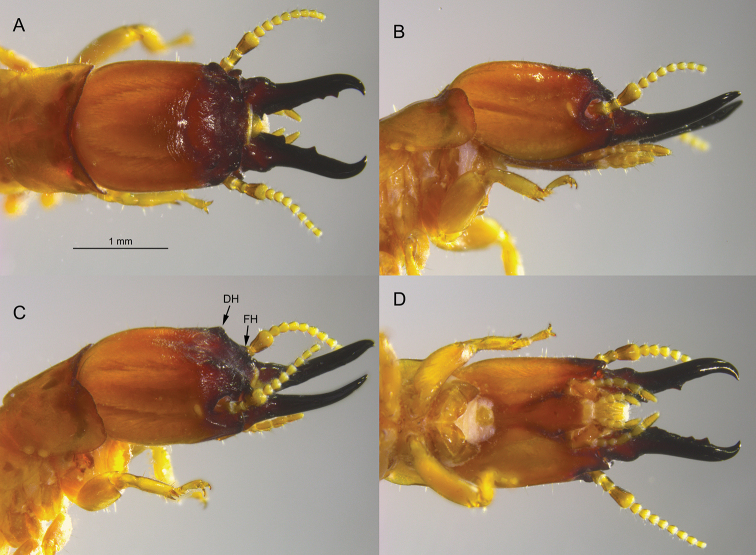
Soldier of *Tauritermes triceromegas* (PA942) **A** dorsal **B** lateral **C** oblique, and **D** ventral views of head and pronotum. DH = dorsal horn and FH = frontal horn.

**Figure 4.**
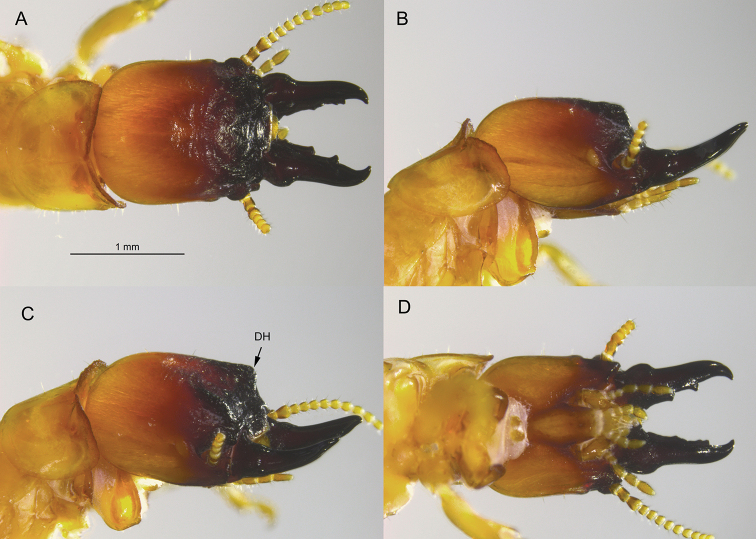
Soldier of *Tauritermes taurocephalus* (BO722) **A** dorsal **B** lateral **C** oblique, and **D** ventral views of head and pronotum. DH = dorsal horn.

The *T. bandeirai* soldier differs from its congeners in that its frons and horns are more rugose, the basal mandible humps are broader and more angular, and the third antennal article is more club-shaped. Only *T. taurocephalus* has a similarly shaped postmentum (Figs 2D, 4D). In *T. triceromegas*, the postmentum is posteriorly elongated (Fig. 3D) and in *T. vitulus* it is posteriorly widened (Araujo and Fontes 1979, fig. 15).

The imago head and pronotum of *T. bandeirai* are mostly unremarkable, except for a relatively large ocellus in comparison with the rather small compound eye. Among kalotermitid genera, the forewing venation is closest to *Incisitermes* Krishna with one diagnostic exception. In *Incisitermes*, the median vein is not sclerotized and its terminus does not closely approach the radial sector (Scheffrahn 2014, fig. 5). In *T. bandeirai*, the distal third of the median vein is sclerotized and closely paralells the radial sector (Fig. 1C). The wing venation of *T. vitulus* is similar to that of *T. bandeirai*, but no sclerotization is reported (Araujo and Fontes 1979, fig. 1).

#### Description.

***Imago*** (Fig. 1A–C, Table 1). Head capsule and pronotum light brown. Compound eye obtusely triangular; ocellus a shade lighter than vertex, very large, and roundly ellipsoid; nearly touching eye margin. Vertex covered with about one dozen short setae. Pronotum about as wide as head capsule; anterior margin weakly emarginate in middle. Pronotum covered with a few dozen setae in middle, lateral margins with about one dozen setae each. Antennae with 15 articles, basal article relative lengths 1>2<3>4. Fore wing with subcosta joining costal margin at about one-eighth of wing length from suture. Radius joining costal margin at one-third wing length; radial sector with about four anterior branches. Median vein becoming lightly sclerotized at distal third as it encroaches near the radial sector. Wing membrane and first 4–5 branches of cubitus lightly pigmented, concolorous with apical radial sector and median veins. Arolium present.

**Table 1.** Measurements (mm) of the *Tauritermes bandeirai* sp. nov. imago.

Character	Females, four colonies (n = 7)		Males, one colony (n = 4)	
	Mean	Range	Mean	Range
Head width, maximum (w/out eyes)	0.8	0.63–0.88	0.81	0.79–0.86
Head width, maximum (with eyes)	0.93	0.70–1.04	0.93	0.91–0.98
Pronotum, maximum width	0.98	0.90–1.08	0.85	0.83–0.90
Eye diameter, maximum	0.33	0.30–0.35	0.29	0.26–0.30
Body length	5.54	4.56–6.32	5.08	4.88–5.36
Right fore wing length	7.7	7.62–7.78	6.56	6.51–6.67
Body length with wings	9.92	9.84–10.00	8.47	8.25–8.73
Number of antennal articles	15	15	–	–

***Soldier*** (Fig. 2A–D, Table 2). Head capsule, in dorsal view (Fig. 2A), dark castaneous brown from postclypeus to antennal carinae, grading to orange at occiput. Head capsule narrowing toward antennal carinae; antennal carinae rugose and visible from above. Frontal ridge V-shaped with deep median cleft. About a dozen fine setae on vertex and genae. Eye spots hyaline, narrowly elliptical. In lateral view (Fig. 2B), dorsal horns (or protuberances) forming a rounded shelf near right angle; frontal horns projecting above base of mandibles. Genal horns slightly posterior to frontal horns, anterior to dorsal horns. Pronotum shield-shaped, wider than head; anterior margin rugose, incised in middle with rounded anterior lobes. In oblique view (Fig. 2C), dorsal and frontal horns rising prominently from frons; horns and frons coarsely rugose. Postclypeus (Fig. 2D) forming elongated, nearly symmetrical hexagon. Third antennal article slightly club-shaped, relative article length 2<3>4=5. Mandibles two-fifths length of head capsule; basal humps robust, rugose; lateral margin of humps parallel. Outside margin of blade nearly straight from above, then curving at right angle at one-fifth from apex; apical tooth thick, marginal dentition weak. Mandibles curve evenly by about 15° in lateral view.

**Table 2.** Measurements of *Tauritermes bandeirai* sp. nov. soldiers (n = 21) from nine colonies.

Character	Mean	Range
Head length to tip of mandibles	2.12	1.95–2.25
Head length to postclypeus	1.42	1.30–1.50
Head width, maximum	1.11	0.95–1.20
Antennal carinae, outside span	1.08	0.98–1.18
Span of dorsal horns	0.82	0.72–0.95
Span of frontal horns	0.89	0.82–0.98
Labrum, maximum width	0.25	0.21–0.32
Pronotum, maximum width	1.11	0.98–1.19
Pronotum, maximum length	0.82	0.70–0.93
Left mandible length to ventral condyle	1.00	0.88–1.10
Postmentum, maximum width	0.37	0.33–0.40
Postmentum, minimum width	0.22	0.18–0.26
Postmentum, length in middle	0.57	0.50–0.68
Head height, excluding postmentum	0.88	0.77–0.96
Third antennal article length	0.13	0.09–0.18
Number of antennal articles	10.58	9–12

#### Type material examined.

***Holotype* soldier**, Brazil: Paraíba, São José da Mata (7.1829, -35.9767); 659 meters A.S.L., 17AUG2000, A. Vasconcellos (AV); one soldier (labelled as holotype, Fig. 2), one soldier and pseudergates (paratypes); University of Florida Termite Collection (UFTC) no. SA499, subsample from Universidade Federal da Paraíba Termite Collection (UPTC) no. 3160.

#### Other material examined.

Brazil: Bahia, Itagiba, Fa. Conjunto S. Luis (-14.2840, -39.8428), 194 m, 18MAR1994, Jan Křeček; one soldier, pseudergates; UFTC SA444. Bahia, Morro do Chapéu (-11.6474, -41.2694), 974 m, 5NOV2015, AV; four soldiers, four imagos, pseudergates; SA504, 7309 (UFTC and UPTC accession numbers respectively). Bahia, Milagres (-11.6473, -39.8333), 700 m, 16MAR2012, AV; four soldiers, pseudergates; 4362. Paraíba, Maturéia (-7.2669, -37.3514); 700 m, 20MAY2000, AV; three soldiers, pseudergates; SA497, 1255. Paraíba, Mamanguape (-6.8386, -35.1261); 33 m, 24JUN2000, AV; two soldiers, pseudergates; SA498, 1799. Paraíba, João Pessoa (-7.1554, -34.8731); 53 m, 20DEC2012, AV; three soldiers, five imagos; SA502, 4747. Paraíba, São José dos Cordeiros, RPPN Faz. Almas (-7.3905, -36.8083), 523 m, 07MAR2003, AV; three soldiers, pseudergates; 4746. Pernambuco, Buíque (-8.5333, -37.2333); 705 m, 16APR2009, AV; two soldiers, one imago, pseudergates; SA500, 3307. Pernambuco, Floresta Tacaratu (-8.6500, -38.0167); 924 m, 29JUN2010, A. A. V. O. Couto; one soldier, two imagos, pseudergates; SA503, 5014. Pernambuco, Igarassu (-7.8371, -35.0006); 129 m, 10MAR2016, A. A. V. O. Couto; two soldiers, pseudergates; SA505, 8512.

#### Distribution.

**Figure 5.**
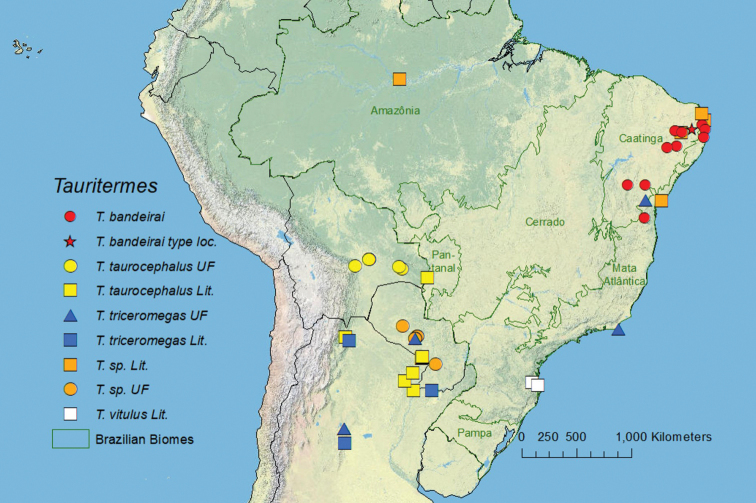
Map of *Tauritermes* from the literature and UFTC. Biomes are shown for Brazil. See Scheffrahn (2019b) for UFTC data and Table 3 for literature references.

(Fig. 5) Northeastern Brazil, Caatinga and Atlantic Forest biomes. *Tauritermes* localities taken from the literature are given in Table 3.

**Table 3.** Localities of *Tauritermes* spp. Taken from the literature and mapped in Fig. 5.

*Tauritermes* sp.	Location	Latitude / Longitude	Reference
*T.* sp.	Brazil: Paraíba	-7.47, -36.87	Vasconcellos et al. (2010)
*T.* sp.	Brazil: Mataraca	-6.48, -34.93	Vasconcellos et al. (2005)
*T.* sp.	Brazil:Amazonas, Manaus	-3.1, -59.97	Dambros et al. (2013)
*T.* sp.	Brazil: “Atlantic forest”	-5.93, -35.18	Souza et al. (2012)
*T.* sp.	Brazil: Bahia, Mata de S. João	-12.97, -38.51	Cancello et al. (2014)
*T.* sp.	Argentina: Picomayo P. Nat.	-25.109, -58.144	Roisin and Leponce (2004)
*T. taurocephalus*	Argentina: Corrientes	-27.49, -58.8	Torales et al. (1997)
*T. taurocephalus*	Brazil: Mato Grosso, Corumbá	-19.02, -57.65	Silvestri (1901)
*T. taurocephalus*	Argentina: Chaco, Captain Solari	-26.8, -59.56	Godoy (2004)
*T. taurocephalus*	Artentina: Formosa, Pres. Irigoyen Dept.	-26.18, -58.85	Godoy (2004)
*T. taurocephalus*	Argentina: Formosa, P. N. Picomayo	-25.066, -58.089	Roisin and Leponce (2004)
*T. taurocephalus*	Argentina: Formosa, P. N. Picomayo	-25.026, -58.097	Roisin and Leponce (2004)
*T. taurocephalus*	Argentina: Salta, Urundel	-23.56, -64.4	Fontes (1998)
*T. taurocephalus*	Argentina: Salta, Urundel	-23.56, -64.4	Fontes (1998)
*T. triceromegas*	Argentina: Cordoba, Cosquin	-31.24, -64.47	Silvestri (1901)
*T. triceromegas*	Argentina: Corrientes, Concepcion	-27.48, -57.3	Torales et al. (1997)
*T. triceromegas*	Argentina: Salta, La Estrella	-23.82, -64.07	Fontes (1998)
*T. vitulus*	Brazil: Santa Catarina, Blumenau	-26.9, -49.1	Araujo and Fontes (1979)
*T. vitulus*	Brazil: Santa Catarina, Itapema	-27.1, -48.6	Araujo and Fontes (1979)

#### Etymology.

Named for Dr. Adelmar Gomes Bandeira, the graduate and post-graduate advisor of AV who died in 2019. Dr. Bandeira was one of the first termitologists to work on termite ecology in the New World.

#### Biology.

The colonies of *T. bandeirai* were collected inside dry trunks in the beginning stages of decomposition (diameter > 3cm) and in dead terminal branches still attached to the trunks, both in areas of Caatinga and Atlantic Forest. In the Caatinga, colonies of *T. bandeirai* were relatively easy to extract from dead terminal branches of *Commiphora leptophloeos* (Mart.) J.B. Gillett (Burseraceae). This tree is also a “hot spot” for collecting other kalotermitids, such as *Cryptotermes*, *Neotermes*, and *Rugitermes*.

Using light traps over a year (December 2017 to November 2018) in a Caatinga area located in the municipality of São José dos Cordeiros, Paraíba-Brazil, the alates of *T. bandeirai* were collected five times; once in December, thrice in January, and once in February. This period represents a transition between the dry and rainy season in the area. For the Atlantic Forest, alates were recorded in wood in March, June, and December.

There are no records of *Tauritermes bandeirai* infestations in buildings, either in urban or agricultural environments. Other *Tauritermes* species infest sound, dry wood (RHS, unpubl.) and are even structural pests (Araujo and Fontes 1979).

## Discussion

The Caatinga and the Atlantic Forest are neighboring domains (Fig. 5), but drastically different in terms of age and environmental conditions. The Caatinga is a semiarid region of northeastern Brazil and is part of the “Seasonally Dry Tropical Forests” (Silva et al. 2017). On the other hand, the Atlantic Forest is distributed along the east coast of South America and is part of the “Tropical Rain Forests” (Morellato and Haddad 2000). Even with such different physiognomies and ecological dynamics, several species of termites, in addition to *T. bandeirai* sp. nov, are found in both domains such as *Heterotermes longiceps* (Snyder, 1924), *Ruptitermes reconditus* (Silvestri, 1901), *Nasutitermes macrocephalus* (Silvestri, 1903), *Microcerotemes indistinctus* Mathews, 1977, among others.

## Supplementary Material

XML Treatment for Tauritermes bandeirai
